# Caught in the Web: A Review of Web-Based Suicide Prevention

**DOI:** 10.2196/jmir.2973

**Published:** 2014-01-28

**Authors:** Mee Huong Lai, Thambu Maniam, Lai Fong Chan, Arun V Ravindran

**Affiliations:** ^1^Faculty of Medicine & Health SciencesDepartment of Psychological MedicineUniversiti Malaysia SarawakKuchingMalaysia; ^2^Department of PsychiatryUniversiti Kebangsaan Malaysia Medical CentreUniversiti Kebangsaan MalaysiaKuala LumpurMalaysia; ^3^Department of PsychiatryUniversity of Toronto and Centre for Addiction and Mental Health (CAMH)Toronto, ONCanada

**Keywords:** suicide prevention, Web-based, Internet

## Abstract

**Background:**

Suicide is a serious and increasing problem worldwide. The emergence of the digital world has had a tremendous impact on people’s lives, both negative and positive, including an impact on suicidal behaviors.

**Objective:**

Our aim was to perform a review of the published literature on Web-based suicide prevention strategies, focusing on their efficacy, benefits, and challenges.

**Methods:**

The EBSCOhost (Medline, PsycINFO, CINAHL), OvidSP, the Cochrane Library, and ScienceDirect databases were searched for literature regarding Web-based suicide prevention strategies from 1997 to 2013 according to the modified PRISMA (Preferred Reporting Items for Systematic Reviews and Meta-Analyses) statement. The selected articles were subjected to quality rating and data extraction.

**Results:**

Good quality literature was surprisingly sparse, with only 15 fulfilling criteria for inclusion in the review, and most were rated as being medium to low quality. Internet-based cognitive behavior therapy (iCBT) reduced suicidal ideation in the general population in two randomized controlled trial (effect sizes, *d*=0.04-0.45) and in a clinical audit of depressed primary care patients. Descriptive studies reported improved accessibility and reduced barriers to treatment with Internet among students. Besides automated iCBT, preventive strategies were mainly interactive (email communication, online individual or supervised group support) or information-based (website postings). The benefits and potential challenges of accessibility, anonymity, and text-based communication as key components for Web-based suicide prevention strategies were emphasized.

**Conclusions:**

There is preliminary evidence that suggests the probable benefit of Web-based strategies in suicide prevention. Future larger systematic research is needed to confirm the effectiveness and risk benefit ratio of such strategies.

## Introduction

Suicide is a common and serious problem worldwide. The World Health Organization reported in 2009 that nearly one million people commit suicide yearly, with 3000 deaths a day or 1 suicide every 40 seconds. Suicide is also the second largest cause of mortality in the 10-24 year age group [1].

The emergence and rapid growth of the digital world and its technology tools has had a tremendous influence on people’s lives. World Internet usage has increased 566.4% from 2000 to 2012 [2]. In particular, there has been an obvious and growing interest both in the negative contribution of the Internet to suicidal behaviors, as well as on its potential usefulness as a tool for prevention [3-9].

There are several lines of evidence suggesting that the Internet may negatively influence suicidal behaviors in certain vulnerable groups. First, there have been clusters of suicides after sensational reporting of suicide cases in the media, referred to as the “Werther effect” or copycat suicides [10-14]. The effect of the Internet in reporting and glorifying suicide may be regarded as comparable to that of other conventional media [5]. Sporadic cases of copycat suicide via the Internet have been reported [4,15,16]. Second, pro-suicide websites and chat rooms often facilitate suicide pacts [17,18] and provide detailed models of lethal methods [15,19]. They also frequently discourage constructive help-seeking behaviors and often exert peer pressure on vulnerable Internet users who are ambivalent about committing suicide [16,20].

The Internet can be viewed as a double-edged tool [5]. While it is accepted that the Internet may be used to trigger and encourage suicidal behavior, its potential as a tool for suicide prevention has been equally recognized [8,9,21,22]. There is good evidence that populations vulnerable to suicide often access Web-based resources. For example, it has been shown that half of the service users of some online suicide prevention programs were clearly suicidal [23]. Similarly, a Web-based study among people with common mental disorders showed that 53.4 % (268/502 participants) reported some degree of suicidal ideation [24] and that suicide threats were more frequent among users of an online support group than among telephone hotline users [25]. Gould et al (2002) studied help-seeking resources for emotional distress in a community sample of adolescents and found that 18.2% (94/519 participants) used the Internet as their help-seeking tool and approximately one in ten of those logged in the Internet was seeking help for their suicidal thoughts [26]. According to Mohd Daud (2005), about a third of high school students from Malaysian schools with high rates of disciplinary problems used the Internet as a help-seeking tool for their emotional problems, out of which 6.4% (7/110 students) cited suicidal thoughts as being the reason for seeking help [27]. Interestingly, it has been noted that a majority of the hits generated by the search engine Google on the word “suicide” in 2005, 2009, and 2012 had a clear suicide-preventive message [28].

Durkee et al (2011) reviewed different pathways by which suicidal risks and prevention efforts are facilitated through the Internet, particularly in young people [8]. Daine et al (2013) performed a systematic review and concluded that Internet use may exert both positive and negative effects on young people at risk of self-harm or suicide [29]. To the best of our knowledge, there is currently no published systematic review focusing specifically on existing Web-based suicide prevention strategies. Therefore, this paper aims to review the available Web-based suicide prevention strategies and evaluate the evidence for their efficacy, benefits, and challenges.

## Methods

**Figure 1:**
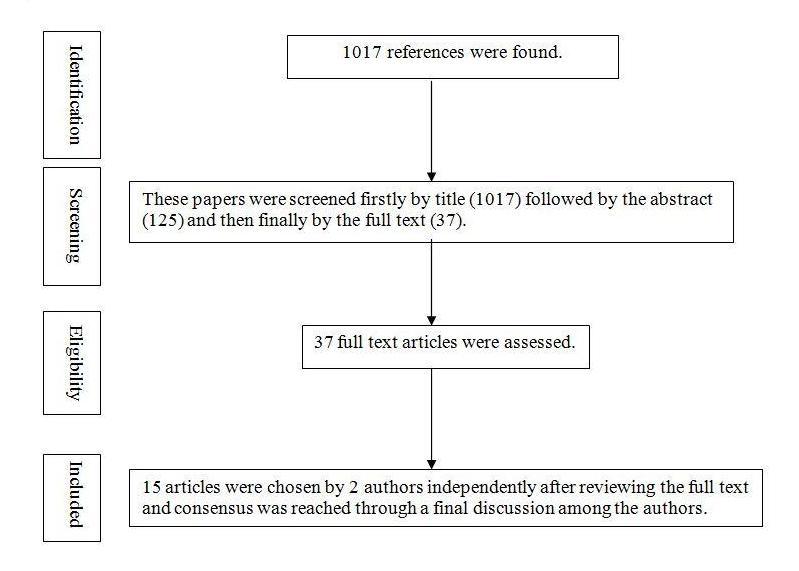
Flow chart of identification, screening, eligibility assessment, and final inclusion of number of articles.

The modified PRISMA (Preferred Reporting Items for Systematic Reviews and Meta-Analyses) statement [30] was used as the methodological approach for this review. The search engines were EBSCOhost (Medline, PsycInfo, CINAHL), OvidSP, the Cochrane Library, and ScienceDirect from 1997-2013. The keywords were *suicide prevention*, *suicide support group*, *suicide help and Internet* or *suicide help and Web-based* or *suicide help online*, either in the title, abstracts, or keywords of the publications. Only English language papers were included. One criterion for inclusion in this review was that the publication should have a clear discussion of efficacy or benefits or challenge of a specific suicide prevention strategy. Letters to the editor and sample descriptions of study protocols alone were excluded, as well as articles that focused solely on the process of training and education on suicide prevention. The quality of primary research papers (not applicable in the case of review papers) was assessed according to the “Standard Quality Assessment Criteria for Evaluating Primary Research Papers from a Variety of Fields” by the Alberta Heritage Foundation for Medical Research [31]. The number of articles that were selected following the PRISMA statement with modification is shown in Figure 1. The initial screening process was done by 1 author (MHL). The selection of 15 articles was based on the independent assessment of the full-text articles by 2 authors (MHL and LFC), and consensus was reached through a final discussion among 3 authors (MHL, LFC, and MT).

## Results

The published literature on good-quality Web-based suicide prevention programs was sparse, with 15 articles (from 2007-2013) fulfilling our criteria as assessed independently by 2 authors. Two articles were narrative type review papers [32,33]. For primary research papers, there were two randomized controlled trials (RCTs) [34,35], but one of the RCTs produced two papers [34,36] that evaluated the efficacy and cost-effectiveness of unguided online cognitive behavioral therapy (CBT)-based self-help to reduce suicidal thoughts. Other primary research papers were one pre- and posttreatment case series [37], two cohort studies [38,39], two cross-sectional studies [40,41], one qualitative study [42], and four descriptive reports [23,43-45]. Based on the quality assessment and ratings of the 13 primary research papers, five papers were rated as high quality, five rated as medium quality, and three rated as low quality (Multimedia Appendix 1).

For the evaluation of the efficacy of the reviewed Web-based suicide prevention strategies, two review papers did not find clear evidence to confirm the efficacy of those strategies. Nevertheless, those strategies seemed promising and had the potential for greater opportunities for suicide prevention [32,33]. Three studies on the effectiveness of interventions based on Internet CBT (iCBT) showed some benefit in reducing suicidal ideation [34,35,37]. Spijker’s (2012) RCT of 6 weeks in a general population sample aimed to test the efficacy and cost-effectiveness of an iCBT-based self-help intervention that consisted of 6 modules. The control group received access to a website with information on suicidality and common treatment options with links to mental health care institutions. The intervention arm showed a significant improvement in suicidal thoughts via a cost-effective way [36], albeit with a small effect size (*d*=0.28) [34]. Christensen et al’s (2013) RCT showed that a 6-week course of iCBT among telephone helpline callers significantly reduced suicidal ideation. However, iCBT with or without telephone call back was not more effective compared to telephone call back alone (participants were contacted for 10 minutes systematically once a week for 6 weeks) or treatment-as-usual control condition (participants could call the crisis line at any time). Effect sizes of iCBT intervention were relatively small ranging from 0.04-0.45 over a 12-month period [35]. Watts et al’s (2012) effectiveness study within a non-controlled clinical audit showed a significant reduction in suicidal ideation regardless of sex and age in a sample of primary health care patients who were given a six-lesson course of iCBT for depression [37].

The other papers mainly described and evaluated the existing real-life Web-based suicide prevention strategies without directly studying their efficacy with objective measurable outcomes. Among them, seven publications anecdotally reported some degree of positive benefit for users. These included cohort and descriptive studies among university students with suicidal behavior who seemed to have improved access to face-to-face mental health services after using the Internet as a means of communication on institution-supported Web programs [38,39,45]. The other two papers reported the formation of support groups; one was qualitative research on understanding the help-seeking experience through an online message board with the theme of “suicide” [42] and the other was a descriptive paper on the model of an online support group [44]. Two other papers reported positive feedback from users who were in crisis [23,40].

One study evaluated the quality of information regarding youth suicide in community accessed websites for youth and found that less than half of such websites contained evidence-based statements [41]. Lester (2008) described the benefits and feasibility of counseling of suicidal individuals via email [43].

The majority of the programs utilized were developed and implemented by four non-governmental organizations (NGOs) [23,42-44], and three others were university-based suicide prevention programs [38,39,45]. Most of the Web-based programs provided information on contributing factors, guidance on seeking help, and information on available resources. In addition, interventional components that included elements of support, counseling, or cognitive therapy were integrated into most of the Web-based programs (Multimedia Appendix 1). The 15 articles have been grouped under the following categories in Multimedia Appendix 1: reviews, RCTs, and descriptive/naturalistic studies.

The literature suggests that three factors—ease of accessibility, degree of anonymity, and the nature of text-based communication—are key components for Web-based suicide prevention strategies. Each of these components provides certain benefits but also poses some challenges (Table 1).

**Table 1:** Benefits and challenges of the main characteristics of Web-based suicide prevention programs.

Characteristics	Benefits	Challenges
Accessibility	Convenient access to information [23]	Unauthorized access may affect the data accuracy; may lead to breaches of confidentiality [43]
	Providing international services across geographical [42,43], cultural, and physical boundaries [43,44]	Limits access for those without resources [43]
		May create inappropriate counseling interventions due to cultural differences [33]
		Logistical difficulties of intercountry referral process [33]
		Lack of formal environment may reduce efficacy of therapy [43]
Anonymity	Alternative to conventional services that may reduce barriers to help-seeking, especially in vulnerable populations seeking anonymity [23,33,39,43]	Risk of non-genuine message postings (estimated <5% of all communications with SAHAR) and accelerating burnout among helpers [23]
	Decreasing inhibitions with regard to self-disclosure and prompting of ventilation [23]	Risk of “flaming effect” with insults and hostile comments affecting at-risk individuals [33]
	Higher assurance of confidentiality in the context of sharing sensitive personal issues, including suicidal thoughts in a supportive online community [42]	Difficulties in sustaining therapeutic alliance with multiple “therapists” online [43]
		Ethical issues of identifying and monitoring quality of therapists [33,43]
Text-based	Writing has therapeutic effect [33]	Safety concern, ie, delayed response to an acute crisis [33,38]
	Flexibility of time in terms of immediate ventilation during crisis [33] and control in timing of sending message [42]	Risk of Internet addiction in clients who write excessively [43]
		Benefits limited to literate clients [43]
		Technical problems that interrupt communication [23]
	Saved text of counselor’s response can be studied by client [33] and also used to monitor and mentor the therapists and data for research [33,43]	Lack of physical cues [33] and non-immediate feedback [43] may impair communication
	Potentially an ideal communication tool for those with hearing or speech impairment and volunteers with physical impairment [43]	Therapists need to undergo training to be familiar with text-based communication [38]
	Resource-saving in terms of ability of experienced therapists in conversing with a few clients simultaneously [23]	The nature of communication that is saved may increase therapist anxiety [43]

## Discussion

### Principal Findings

This review confirms the paucity of strong evidence-based literature on the effectiveness of Web-based suicide prevention strategies. After the quality assessment of the primary research papers, a majority (8/13) of them were rated as medium to low quality. The interpretation of published data was difficult due to methodological constraints such as the heterogeneous nature of the strategies, target population, and the lack of systematic outcome evaluations. Greater attention was paid to papers of higher quality.

To date, two published RCTs examined the effectiveness of iCBT-based interventions in reducing suicidal thoughts in a general population [34,35]. Both demonstrated positive benefits of iCBT with some suggestion of changes in hopelessness [34] and depressive symptoms [35] as possible mediating mechanisms, as well as some evidence of cost-effectiveness [36]. However, effect sizes were small and Christensen’s (2013) study failed to show superiority of iCBT with or without telephone call back compared to telephone call back alone or treatment-as-usual controls [35]. iCBT may be effective in reducing suicidal ideation in a clinically depressed population, although the level of evidence is less robust in view of the lack of controlled studies [37]. Whether such positive benefits on suicidal ideation can be sustained over time warrants longer-term studies. Well-controlled studies examining outcome after intervention for severe suicidal behavior, such as attempted or completed suicide, do not exist, which highlights the need for such studies in the future.

Nevertheless, there is some preliminary evidence from descriptive reports that the Internet may serve as a good step for individuals with suicidal thoughts to gain better access to face-to-face mental health care services [38,39,45]. The anonymous nature of Internet communication is likely to help reduce barriers in accessing mental health services due to stigma, particularly among adolescents, celebrities [23], and medical personnel [39].

The online suicide prevention approaches reviewed were automated (such as in iCBT), informative, or interactive in nature and mostly comprised a combination of the above. The quality of information, as well as the evidence base for their benefit, appear to vary between programs. For example, evaluated the quality of information on such resource sites and found that less than half of the information on the Canadian suicide prevention websites was truly evidence-based [41]. The target groups for most of the suicide prevention programs were also not often specified. A notable exception was the program outlined by Feigelman et al (2008). It described the demographic and loss-related characteristics of parents in an Internet suicide survivor support group [40].

Interactive approaches of Web-based suicide prevention often target an individual or alternatively lead to the formation of a support group. The means to reach an individual can be via email [23,38,39,43,44], chat software [23,44], or advertisements on websites and in newspapers [23]. Creation of an online support group is often achieved through online forums [23], chat rooms [44], social networking [45], or bulletin boards [42]. User feedback in the published studies indicate that, in safe and successful programs, the support group is created through supervised chat rooms or through a forum involving trained volunteers [42]. In the absence of such supervision, chat rooms or forums are thought to be potentially harmful [33].

The appealing characteristics of Web-based suicide prevention strategies (ie, ease of accessibility, greater degree of anonymity, and component of text-based communication) give rise to the benefits of promoting help-seeking behavior and potentially accessing hard-to-reach at-risk groups [23,33,39,43]. However, those characteristics also pose some potential challenges such as the risk of breach of confidentiality [43], inappropriate counseling derived from cultural differences [33], hostile comments that affect at-risk individuals [33], fake messages that accelerate burnout among volunteers in online groups [23], and safety concerns in an acute crisis [33,38]. Therefore, Miller (2009) suggested that the development of consumer guidelines regarding the safer use of the Internet is paramount [33]. However, systematic research confirming these challenges is still lacking (Table 1).

There is also general agreement [21,32] that the evaluation of Web-based suicide prevention strategies has many challenges. The anonymity of the participants makes valid sampling and measurement outcome more difficult. Therefore, more robust design in fully utilizing the benefits and managing the challenges of the characteristics of the Internet as a communication tool is needed for future study. Further large-scale systematic research is needed to confirm the evidence base for the effectiveness as well as the risk-benefit ratio of these Web-based strategies. According to Boyce (2010), future research also should aim “to understand the path that people with suicidal thoughts travel online, and to work out when and how to intervene” (p. 1890). He further recommended “a mixture of both quantitative and qualitative methods, examining both overall online trends and individual views” (p. 1890) [22]. Efficacy and cost effectiveness of Web-based intervention to reduce suicidality, including comparison between Web-based, telephone hotlines, and face-to-face interventions are other areas for future research.

### Limitations

This review has several limitations. The number of publications available and included was small and mostly non-RCT in nature. Furthermore, the pooled information was difficult to evaluate due to the heterogeneous nature of the population, differences in design, and measurement methods. A more detailed search strategy that included studies identifying interventions for depression with suicidal outcomes that were not explicitly mentioned in the title or abstract would have been more comprehensive. In addition, non-English publications were not included in the review. Other publication biases included the following search omissions: grey literature, and contact with experts in the field to find additional unpublished studies. As mentioned, most of the studies were uncontrolled and unblinded, therefore subject to the risk of observer bias in the reported outcomes. In the case of RCTs, blinding of participants was not possible due to the nature of the active intervention delivered, that is, iCBT. Hence, there might be a tendency towards more positive outcomes based on the self-report of participants receiving iCBT due to favorable expectations towards receiving active intervention versus treatment-as-usual control conditions.

### Conclusions

In conclusion, there appears to be preliminary evidence that suggests the probable benefit of Internet-based suicide prevention strategies as novel and cost-effective interventions. In particular, iCBT may serve as a means to reduce suicidal ideation. Web-based approaches seem to be advantageous in terms of potentially reaching out to populations at-risk for suicidal behavior for whom conventional methods have limited accessibility due to stigma, physical or psychological limitations, or geographical location. Future research is needed to elucidate the impact of and strategies to overcome the potential challenges such as issues of confidentiality, safety concerns in acute crisis, and feasibility of resources, among others. Findings from such studies would further clarify the risk-benefit ratio in terms of implementation of Web-based suicide prevention strategies as potential adjuncts or alternatives to mainstream interventions in the future.

## Multimedia Appendix 1

Web-based suicide prevention strategies—summary of the included articles.

## Abbreviations

CBT: cognitive behavioral therapy
CINAHL: Cumulative Index to Nursing and Allied Health Literature
iCBT: Internet cognitive behavioral therapy
PRISMA: Preferred Reporting Items for Systematic Reviews and Meta-Analyses
RCT: randomized controlled trial

## References

[1] WHO Media Centre (2009). Suicide risk high for young people.

[2] Internet World Stats (2012). World Internet Users and Population Statistics.

[3] Mehlum L (2006). The Internet, Suicide, and Suicide Prevention. Crisis: The Journal of Crisis Intervention and Suicide Prevention.

[4] Alao AO, Soderberg M, Pohl EL, Alao AL (2006). Cybersuicide: review of the role of the Internet on suicide. Cyberpsychol Behav.

[5] Tam J, Tang WS, Fernando DJ (2007). The Internet and suicide: A double-edged tool. Eur J Intern Med.

[6] Biddle L, Donovan J, Hawton K, Kapur N, Gunnell D (2008). Suicide and the Internet. BMJ.

[7] Recupero PR, Harms SE, Noble JM (2008). Googling Suicide. J Clin Psychiatry.

[8] Durkee T, Hadlaczky G, Westerlund M, Carli V (2011). Internet pathways in suicidality: a review of the evidence. Int J Environ Res Public Health.

[9] Collings S, Niederkrotenthaler T (2012). Suicide prevention and emergent media: surfing the opportunity. Crisis.

[10] Gould MS (2001). Suicide and the media. Ann NY Acad Sci.

[11] Niederkrotenthaler T, Sonneck G (2007). Assessing the impact of media guidelines for reporting on suicides in Austria: interrupted time series analysis. Aust N Z J Psychiatry.

[12] Insel BJ, Gould MS (2008). Impact of modeling on adolescent suicidal behavior. Psychiatr Clin North Am.

[13] Niederkrotenthaler T, Voracek M, Herberth A, Till B, Strauss M, Etzersdorfer E, Eisenwort B, Sonneck G (2010). Role of media reports in completed and prevented suicide: Werther v. Papageno effects. Br J Psychiatry.

[14] Sisask M, Värnik A (2012). Media roles in suicide prevention: a systematic review. Int J Environ Res Public Health.

[15] Lee DT, Chan KP, Yip PS (2005). Charcoal burning is also popular for suicide pacts made on the Internet. BMJ.

[16] Becker K, Mayer M, Nagenborg M, El-Faddagh M, Schmidt MH (2004). Parasuicide online: Can suicide websites trigger suicidal behaviour in predisposed adolescents?. Nord J Psychiatry.

[17] Rajagopal S (2004). Suicide pacts and the Internet. BMJ.

[18] Crump A (2006). Suicide in Japan. The Lancet.

[19] Naito A (2007). Internet Suicide in Japan: Implications for Child and Adolescent Mental Health. Clinical Child Psychology and Psychiatry.

[20] Becker K, Schmidt MH (2005). When Kids Seek Help On-Line: Internet Chat Rooms and Suicide. Reclaiming Children and Youth: The Journal of Strength-based Interventions.

[21] Luxton DD, June JD, Fairall JM (2012). Social media and suicide: a public health perspective. Am J Public Health.

[22] Boyce N (2010). Pilots of the future: suicide prevention and the Internet. The Lancet.

[23] Barak A (2007). Emotional support and suicide prevention through the Internet: A field project report. Computers in Human Behavior.

[24] Hemelrijk E, van Ballegooijen W, Donker T, van Straten A, Kerkhof A (2012). Internet-based screening for suicidal ideation in common mental disorders. Crisis.

[25] Gilat I, Shahar G (2007). Emotional first aid for a suicide crisis: comparison between Telephonic hotline and Internet. Psychiatry.

[26] Gould MS, Munfakh JL, Lubell K, Kleinman M, Parker S (2002). Seeking help from the Internet during adolescence. J Am Acad Child Adolesc Psychiatry.

[27] Mohd Daud T (2005). [Unpublished dissertation for the degree of Master of Medicine(Psychiatry)]. Help seeking behaviour using the Internet: a study among sixteen year old Form Four secondary school students from high risk schools in Kuala Lumpur.

[28] Westerlund M, Hadlaczky G, Wasserman D (2012). The representation of suicide on the Internet: implications for clinicians. J Med Internet Res.

[29] Daine K, Hawton K, Singaravelu V, Stewart A, Simkin S, Montgomery P (2013). The power of the web: a systematic review of studies of the influence of the Internet on self-harm and suicide in young people. PLoS One.

[30] Moher D, Liberati A, Tetzlaff J, Altman DG, PRISMA Group (2009). Preferred reporting items for systematic reviews and meta-analyses: the PRISMA statement. PLoS Med.

[31] Kmet LM, Lee RC, Cook LS Standard quality assessment criteria for evaluating primary research papers from a variety of fields.

[32] Krysinska K, De Leo D (2007). Telecommunication and Suicide Prevention: Hopes and Challenges for the New Century. OMEGA: The Journal of Death and Dying.

[33] Miller K (2009). The potential role of the Internet in suicide prevention. Counselling, Psychotherapy, and Health.

[34] van Spijker BA (2012). Reducing the burden of suicidal thoughts through online self-help.

[35] Christensen H, Farrer L, Batterham PJ, Mackinnon A, Griffiths KM, Donker T (2013). The effect of a Web-based depression intervention on suicide ideation: secondary outcome from a randomised controlled trial in a helpline. BMJ Open.

[36] van Spijker BA, Majo MC, Smit F, van Straten A, Kerkhof AJ (2012). Reducing suicidal ideation: cost-effectiveness analysis of a randomized controlled trial of unguided Web-based self-help. J Med Internet Res.

[37] Watts S, Newby JM, Mewton L, Andrews G (2012). A clinical audit of changes in suicide ideas with Internet treatment for depression. BMJ Open.

[38] Haas A, Koestner B, Rosenberg J, Moore D, Garlow SJ, Sedway J, Nicholas L, Hendin H, Mann JJ, Nemeroff CB (2008). An interactive Web-based method of outreach to college students at risk for suicide. J Am Coll Health.

[39] Moutier C, Norcross W, Jong P, Norman M, Kirby B, McGuire T, Zisook S (2012). The suicide prevention and depression awareness program at the University of California, San Diego School of Medicine. Acad Med.

[40] Feigelman W, Gorman BS, Beal KC, Jordan JR (2008). Internet support groups for suicide survivors: a new mode for gaining bereavement assistance. Omega (Westport).

[41] Szumilas M, Kutcher S (2009). Teen suicide information on the Internet: a systematic analysis of quality. Can J Psychiatry.

[42] Greidanus E, Everall RD (2010). Helper therapy in an online suicide prevention community. British Journal of Guidance & Counselling.

[43] Lester D (2009). The Use of the Internet for Counseling the Suicidal Individual: Possibilities and Drawbacks. OMEGA--Journal of Death and Dying.

[44] Gilat I, Shahar G (2009). Suicide prevention by online support groups: an action theory-based model of emotional first aid. Arch Suicide Res.

[45] Manning J, Vandeusen K (2011). Suicide prevention in the dot com era: technological aspects of a university suicide prevention program. J Am Coll Health.

