# Supplementary material for: Caught in the Web: A Review of Web-Based Suicide Prevention
Source: J Med Internet Res. 2014 Jan 28;16(1):e30. doi: 10.2196/jmir.2973 (PMC3936329; doi:10.2196/jmir.2973)
Supplement: Supplementary file 1 [file jmir_v16i1e30_app1.pdf]

Multimedia Appendix 1. Web-based suicide prevention strategies—Summary of the included articles.

Sources	Design	Organization	Aims and methods	Findings	Quality
**Reviews**					
Krysinska and Leo (2007) [32]	Review	N/A	Overview of telecommunication-based strategies (Internet and telephone)	No clear evidence confirming efficacy of telecommunication-based strategies<br>Approaches seemed promising in increasing accessibility to services for suicidal individuals and raising public awareness	N/A
Miller (2009) [33]	Review	N/A	Discussion of potential role of Internet in suicide prevention through information provision, counseling, and chat rooms	Potential risk of Internet providing easy access to unregulated information<br>Internet counseling not recommended for those at high or immediate risk of suicide without availability of close and intensive monitoring<br>Despite a sense of community in chat rooms, limited avenue to learn new ways to cope<br>Professionally facilitated groups more effective in suicide prevention	N/A
**Randomized-controlled trials**					
Spijker (2012) [34]; Spijker et al (2012) [36]	RCT	N/A	Test effectiveness of unguided iCBT-based self-help to reduce suicidal thoughts and evaluate cost-effectiveness in general population sample (N=236)	For each intervention responder, annual cost savings was €34,727 (US $41,325) compared to controls, and absenteeism improved at work<br>Intervention group (N=116) showed two-fold reduction in suicidal ideation with small effect sizes (d=0.28) at 6 weeks posttest compared to controls; effects maintained at 3-month follow-up<br>Limitations: self-report measures, outcome measures based on short duration	High

Christensen et al (2013) [35]	RCT	Lifeline Australian 24-hour telephone counseling service	Evaluate the effectiveness of a 6-week iCBT intervention (MoodGym) on suicide ideation among telephone helpline callers (N=115)	Data from secondary outcome (suicide ideation) of RCT on iCBT for depression<br>Suicide ideation significantly reduced in all 4 intervention arms (1) iCBT alone, (2) iCBT & telephone call back, (3) telephone call back alone, (4) treatment as usual control<br>iCBT with or without telephone call back not superior to (3) or (4)<br>Resolving depression significantly associated with improvement in suicide ideation<br>Small effect sizes of iCBT (0.04-0.45) up to 1 year	High
**Descriptive/Naturalistic studies**					
Watts et al (2012) [37]	Pre-treatment/ posttreatment case series (effectiveness study within quality assurance framework)	Primary care	Examine suicidal ideation in depressed patients (n=299) who received 6 sessions of iCBT	Suicidal thought (measured by item 9 on the Patient Health Questionnaire) was common (54%) prior to intervention and reduced to 30% post-intervention; reduction was significant<br>No control group	Medium
Haas et al (2008) [38]	Cohort	American Foundation for Suicide Prevention (AFSP)	College Screening Project for suicidal students via an interactive Web-based method	1162 (8% of invited participants) completed screening and 981 (84.4%) were identified as at risk<br>Majority subsequently evaluated (190) and entered treatment (132) did so because of outreach<br>Anecdotal report of lower incidence of suicide in a university setting after Web-based screening	Medium
Moutier et al (2012) [39]	Cohort	University of California, San Diego (UCSD) Suicide Prevention and Depression Awareness Program	1. Web-based, anonymous confidential screening, assessment, and referral program (AFSP)<br>2. Face-to-face education to medical students, and physicians on depression/suicide	374 individuals (13% of those receiving email invitation) completed screens, 27% at high risk for depression or suicide and 13% referred for care<br>12/17 respondents who had first session with counselor credited the online screening program for getting help	Medium

Feigelman et al (2008) [40]	Cross-sectional	Suicide survivor support group	Compare the demographic and loss-related characteristics of parents in Internet (N=104) and face-to-face suicide survivor support groups (N=297)	35% in Internet group compared to 23% from face-to-face group reported suicidal ideation sometimes or more often during the last year<br>10% more Internet affiliates (17% compared to 7%) reported having a suicide plan<br>64% of respondents from Internet group indicated that helping advance goals of suicide prevention and better mental health resources availability as important features of their support group<br>14 anecdotal positive comments in Internet survey in combating depression and suicidal wishes	Medium
Szumilas and Kutcher (2009) [41]	Cross-sectional	N/A	Evaluate the evidence on youth suicide risk factors and prevention strategies from top 20 information websites	Only 44.2% statements were supported by evidence<br>Crediting and recommendation to health consultation increased odds of statements being supported by evidence	High
Barak (2007) [23]	Descriptive	SAHAR (Support and Listening on the Net)	1. Hebrew website<br>2. Personal communication: Chat/email<br>3. Group communication: Online forum/chat	Around 1000 personal contacts per month and half of them are suicidal<br>More than 100 rescue operations of attempters<br>Support/referral<br>Appreciation/positive feedback from users	Medium
Lester (2008) [43]	Descriptive	1. Befrienders (Samaritans)<br>2. SAHAR (Primary source Barak, 2007)	Counseling via Internet	Received 15,309 emails in 1998 to 64,000 in 2001; 50% suicidal in nature.<br>Described feasibility and benefits of email counseling with reply within 24 hours	Low
Gilat and Shahar (2009) [44]	Descriptive	Israeli Assoc'n for Emotional First Aid (ERAN)	1. Online support group via moderators<br>2. Personal chat<br>3. Email	Help-promoting environment for suicidal individuals may be generated; single positive comment from a user	Low
Manning and VanDeusen	Descriptive	Western Michigan University's	1. Online training course<br>2. Website	Over 7000 hits on website<br>Networks identified 1550 "friends"	Low

(2011) [45]		Suicide Prevention Program (SPP)	3. Two social networking website profiles	Positive feedback from students but low participation on online training course<br>Increased frequency of presentation with suicidal ideation for counseling since implementation	
Greidanus and Everall (2010) [42]	Qualitative research (content analysis)	Community suicide prevention agency	Analyze ‘suicide’-themed online message board to explore experiences of help-seeking process	A positive online mutual-help group monitored by trained volunteers for those users was developed<br>Help-seekers who benefited provided peer support to others	High
